# Early administration of fibrinogen concentrate is associated with improved survival among severe trauma patients: a single-centre propensity score-matched analysis

**DOI:** 10.1186/s13017-020-0291-9

**Published:** 2020-01-14

**Authors:** Yuki Itagaki, Mineji Hayakawa, Kunihiko Maekawa, Tomoyo Saito, Akira Kodate, Yoshinori Honma, Asumi Mizugaki, Tomonao Yoshida, Takayoshi Ohyasu, Kenichi Katabami, Takeshi Wada

**Affiliations:** 1 Emergency and Critical Care Center, Sapporo City General Hospital, 1-1 Nishi13, Kita 11, Kita-ku, Sapporo, Hokkaido 060-8604 Japan; 2 Department of Emergency Medicine, Hokkaido University Hospital, Sapporo, Japan

**Keywords:** Cryoprecipitate, Fibrinogen, Fibrinogen concentrate, Fresh frozen plasma, Trauma-induced coagulopathy

## Abstract

**Background:**

Fibrinogen plays an important role in haemostasis during the early phase of trauma, and low fibrinogen levels after severe trauma are associated with haemostatic impairment, massive bleeding, and poor outcomes. Aggressive fibrinogen supplementation may improve haemostatic function, as fibrinogen levels deteriorate before other routine coagulation parameters in this setting. Therefore, we evaluated whether early administration of fibrinogen concentrate (FC) was associated with improved survival in severe trauma patients.

**Methods:**

This single-centre retrospective study evaluated patients with severe trauma (injury severity score ≥ 16) who were admitted to our emergency department between January 2010 and July 2018. The exclusion criteria included age < 18 years, cardiac arrest before emergency department arrival, cervical spinal cord injury not caused by a high-energy accident, and severe burn injuries. The FC and control groups included trauma patients who received and did not receive FC within 1 h after emergency department arrival, respectively. Propensity scores were used to balance the two groups based on the trauma and injury severity score (TRISS), heart rate at emergency department admission, and age. The primary outcome was the in-hospital survival rate.

**Results:**

The propensity scoring model had a *c*-statistic of 0.734, the Hosmer-Lemeshow chi-squared value was 7.036 (degrees of freedom = 8), and the non-significant *p* value of 0.533 indicated a good model fit. The propensity score matching created 31 matched pairs of patients, who had appropriately balanced characteristics. The FC group had a significantly higher in-hospital survival rate than the control group (log-rank *p* = 0.013). The FC group also used significantly higher amounts of red blood cells and fresh frozen plasma within 6 h after emergency department admission. However, the two groups had similar transfusion amounts between 6 and 24 h after emergency department admission.

**Conclusions:**

The present study revealed that early FC administration was associated with a favourable survival rate among severe trauma patients. Therefore, FC may be useful for the early management of trauma-induced coagulopathy and may improve outcomes in this setting.

## Background

Trauma remains a major cause of death [1, 2], which is primarily related to uncontrolled bleeding during the early phase of trauma [3]. Traumatic haemorrhage may be exacerbated by coagulopathy (i.e. trauma-induced coagulopathy). Although the pathophysiology of trauma-induced coagulopathy remains incompletely understood [4–8], we speculate that it is generated by the following mechanisms: (1) coagulation activation, (2) hyperfibrino(geno)lysis, and (3) consumption coagulopathy [5, 6]. Coagulation activation caused by massive tissue injuries cause excessive thrombin generation, which leads to the fibrinogen consumption. Hyperfibrino(geno)lysis is caused by the acute release of tissue-plasminogen activator, which is induced by tissue hypoperfusion and massive tissue injuries-induced coagulation activation. Various coagulation factors and platelets are consumed by coagulation activation and hyperfibrino(geno)lysis. Nevertheless, trauma-induced coagulopathy is often clearly present on emergency department (ED) arrival and is associated with massive haemorrhage, increased transfusion needs, and a high mortality rate [9–14]. Unfortunately, in patients with severe trauma, haemostatic impairment is worsened by haemodilution, hypothermia, and acidosis during the early phases of treatment [5, 7, 15, 16]. Therefore, better management of trauma-induced coagulopathy is needed to improve the outcomes of these patients.

Fibrinogen plays an important role in haemostasis during the early phase of trauma [16–22], as low fibrinogen levels impair the firmness of the fibrin clots that help to control haemostasis. Fibrinogen also accelerates platelet aggregation [6, 23, 24], and many studies have indicated that low fibrinogen levels at ED arrival are associated with haemostatic impairment, massive bleeding, and poor outcomes in patients with severe trauma [11–14, 16, 24–28]. Furthermore, fibrinogen levels deteriorate faster than other haemostatic components during the early phase of severe trauma [6, 11, 27, 29]. Therefore, decreased fibrinogen levels are an important marker for trauma-induced coagulopathy, and fibrinogen supplementation is needed to help maintain haemostatic function [6, 16]. Recent European guidelines have suggested that fibrinogen concentrations should be maintained at > 1.5–2.0 g/L in patients with severe trauma [30], and there is increasing awareness that fibrinogen concentrate (FC) can be effective for managing massive haemorrhage in these patients. Fibrinogen supplementation can be achieved by using fresh frozen plasma (FFP) and cryoprecipitate [31]. However, FFP must be thawed via a time-consuming process [32], and ABO compatibility must be confirmed before administering FFP [33]. Certain trauma centres have recently begun early coagulation factor supplementation using pre-thawed FFP; however, thawed plasma has a short shelf-life and must be discarded if it is not used [34]. While cryoprecipitate contains factor VIII, factor XIII, and von Willebrand factor (unlike FC), cryoprecipitate also requires thawing before administration [26] and carries a risk of viral infection, similar to FFP [35, 36]. Therefore, although aggressive fibrinogen replacement therapy using FFP or cryoprecipitate provides favourable outcomes [17, 37], this benefit must be balanced with the immediate availability and rapid administration of FC, which does not require thawing or confirmation of ABO compatibility [38]. Furthermore, FC administration may increase plasma fibrinogen levels more easily than FFP [31] and may produce a greater increase in fibrinogen levels more rapidly than both FFP and cryoprecipitate [39].

Several reports have indicated that FC administration is effective for patients with severe trauma [18, 38, 40, 41]. For instance, Wafaisade et al. retrospectively examined the effects of FC administration and reported that it helped improve the short- and not long-term mortality rate [18]. Nevertheless, their FC group included patients who were treated in the ED and intensive care unit; this partially obscured the effects of early FC administration [18]. A single-centre randomised controlled trial (RCT) using real-time thromboelastometry also revealed that relative to FFP, coagulation factor concentrates (including FC, prothrombin complex concentrate, and factor XIII concentrate) helped improve outcomes in patients with severe trauma [41]. However, that trial failed to clarify how the patients’ outcomes varied according to the use of FC, prothrombin complex concentrate, and factor XIII concentrate [41]. Akbari et al. also performed a single-centre RCT and reported that patients with severe trauma who received FC had a significantly lower mortality rate and shorter duration of hospitalisation than those who received FFP and the control group [38]. However, that report failed to clearly describe the timing of FC administration [38]. Therefore, to the best of our knowledge, no studies have specifically examined the early administration of FC in patients with severe trauma. The present study aimed to determine whether this strategy improved survival, based on a propensity score-matched analysis.

## Methods

### Patient selection and data collection

This single-centre retrospective study evaluated electronic medical records from a tertiary emergency and critical care centre (Hokkaido University Hospital). The study protocol was approved by our institutional review board, and the requirement for informed consent was waived owing to the retrospective design.

Adult patients with severe trauma (injury severity score ≥ 16) who were directory admitted to our ED between January 2010 and July 2018 were eligible for inclusion. Patients were excluded based on the following criteria: (a) age < 18 years, (b) cardiac arrest before ED arrival, (c) cervical spinal cord injury not caused by a high-energy accident, and (d) severe burn injuries. The records of eligible patients were searched to collect data regarding trauma severity, laboratory test results from ED arrival, clinical characteristics, treatments, transfusion amounts, and patient outcomes.

### Definitions

The patients were divided into an FC group (received FC within 1 h after ED arrival) and a control group (no FC or FC received at 1–24 h after ED arrival). The decision to administer FC, its timing, and the FC amount were fully at the discretion of the attending physicians. The administered FC was commercially available freeze-dried human fibrinogen (Fibrinogen HT i.v. 1 g “JB”, Japan Blood Products Organization, Tokyo, Japan). On sub-group analysis, severe brain injury was defined as injury with a head abbreviated injury scale (AIS) of ≥ 3.

### Statistical analysis

Propensity score matching was used to balance the groups’ characteristics and clinical variables. The propensity scores for early FC administration were estimated using a logistic regression model, based on the trauma and injury severity score, heart rate at ED admission, and age, all of which are related to the early administration of FC. Patients with and without early FC administration were then matched 1:1 based on their propensity scores, using the nearest neighbour method without replacement, and a calliper width of 0.2 standardised deviations for the propensity score. We used the standardised difference to evaluate the covariate balances after the propensity score matching, with absolute standardised differences of > 0.1 considered indicative of a meaningful imbalance. The two groups were then compared using the Mann-Whitney *U* and chi-squared tests, as appropriate. In-hospital survival outcomes were compared using the Kaplan-Meier method and log-rank tests.

In the two sub-groups, namely, patients with blunt trauma and with severe brain injury, additional analyses were performed using the same methods.

All analyses were performed using SPSS software (version 25; IBM Japan, Tokyo, Japan). All reported *p* values were two-tailed, and differences were considered statistically significant at *p* values of < 0.05.

## Results

**Fig. 1:**
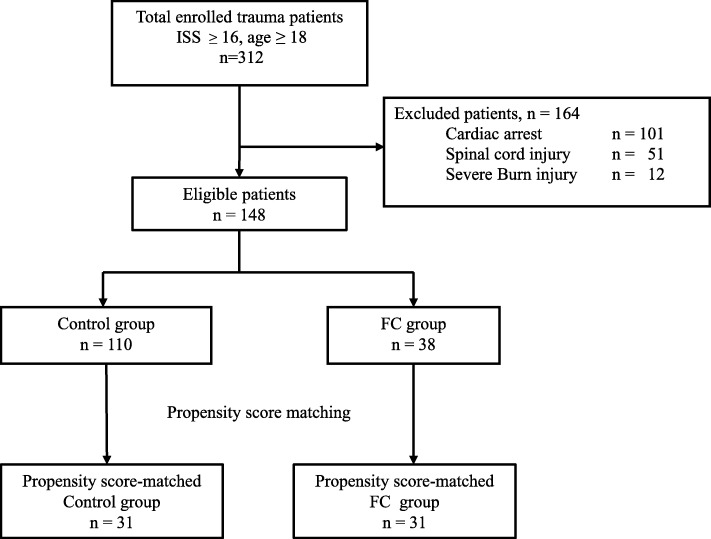
Study flowchart. The fibrinogen concentrate (FC) group included trauma patients who received FC within 1 h after their emergency department admission. The control group included patients who did not receive FC within 1 h after emergency department admission. ISS, injury severity score

During the study period, 480 patients with severe trauma were directly transferred to our ED from the accident site. After the exclusion of ineligible patients, 148 eligible patients were divided into the FC group (38 patients) and the control group (110 patients) (Fig. 1). The patients’ overall characteristics are shown in Table 1; it shows that the FC group had a significantly higher critical status on ED admission. The FC group included trauma patients who received FC within 1 h after ED arrival (*n* = 38), and the control group included 110 patients who did not receive FC within 1 h after ED arrival (39/110 received FC within 1–24 h after admission and 71/110 did not receive FC).

**Table 1:** Characteristics of the patients

	Control group *n* = 110	FC group *n* = 38	*p* value
Age (year)	54 (39–71)	53 (32–72)	0.565
Gender, male	86 (78.2%)	20 (52.6%)	0.005
Blunt trauma	106 (96.4%)	36 (94.7%)	0.647
Mechanism of the injury			
Traffic accident (in the car)	29 (26.4%)	13 (4.2%)	0.313
Traffic accident (pedestrian)	29 (26.4%)	13 (34.2%)	
Fall	39 (35.5%)	10 (26.3%)	
Invert	6 (5.5%)	0 (0.0%)	
Stab wound/cutting	3 (2.7%)	2 (5.3%)	
Others	4 (3.6%)	0 (0.0%)	
Injury to the admission to ED			
0–30 (min)	12 (10.9%)	9 (23.7%)	0.138
30–60 (min)	64 (58.2%)	21 (55.3%)	
60–90 (min)	21 (19.1%)	3 (7.9%)	
90– (min)	13 (11.8%)	5 (13.2%)	
Revised trauma score	6.72 (5.03–7.84)	5.68 (4.09–6.08)	< 0.001
Probability of survival	0.842 (0.630–0.943)	0.567 (0.230–0.832)	< 0.001
Vital signs on the admission to ED			
Heart rate (per min)	86 (72–105)	110 (86–120)	0.010
Glasgow Coma Scale	11 (6–14)	6 (3–13)	0.035
Systolic Blood Pressure (mmHg)	120 (99–151)	93 (68–137)	0.014
Respiratory rate (per min)	22 (16–25)	21 (17–30)	0.489
Injury severity score	25 (20–32)	34 (25–41)	< 0.001
Abbreviated injury scale			
Head/neck	4 (1–5)	4 (0–5)	0.264
AIS face	0 (0–0)	0 (0–1)	0.482
AIS chest	3 (0–4)	3 (0–4)	0.104
AIS abdomen	0 (0–2)	0 (0–3)	0.065
AIS extremity	1 (0–3)	2 (0–3)	0.047
AIS external	1 (0–1)	1 (0–1)	0.082
Blood gas analysis			
pH	7.35 (7.30–7.38)	7.24 (7.11–7.35)	< 0.001
Base deficit (mmol/L)	2.5 (0.3–5.4)	6.95 (2.7–14.63)	< 0.001
Lactate (mmol/L)	3.2 (2.3–4.7)	5.8 (3.6–10.0)	< 0.001
Laboratory tests			
Platelet (× 10^3^/μL)	200 (147–235)	193 (146–234)	0.632
PT-INR	1.06 (0.98–1.158)	1.22 (1.12–1.40)	< 0.001
Fibrinogen (mg/dL)	193 (159–235)	156 (137–219)	0.034
FDP (μg/mL)	76.4 (30.0–165.0)	99.5 (45.0–188.0)	0.349
D-dimer (μg/mL)	47.8 (19.5–104.2)	58.3 (28.3–104.9)	0.633

*FC* fibrinogen concentrate, *ED* emergency department, *AIS* abbreviated injury scale, *PT-INR* prothrombin time-international normalised ratio, *FDP* fibrin/fibrinogen degradation products

The propensity score model had a *c*-statistic of 0.734, which indicated good discrimination between the patients assigned to the FC and control groups. The Hosmer-Lemeshow chi-squared value was 7.036 (degrees of freedom = 8), and the non-significant *p* value of 0.533 indicated a good model fit. The propensity score matching process ultimately selected 31 patients from each group, and the characteristics of the matched patients are shown in Table 2. The two groups had generally well-balanced characteristics including the probability of survival, which provides a comprehensive assessment of trauma severity. Most imbalanced variables were more severe in the FC group than in the control group.

**Table 2:** Characteristics of the propensity score-matched patients

	Control group *n* = 31	FC group *n* = 31	Matched standardised difference	*p* value
Age (years)	55 (36–72)	53 (32–74)	− 0.006	0.978
Gender, male	25 (80.6)	19 (61.3)	− 0.435	0.093
Blunt trauma	29 (93.5%)	29 (93.5%)	< 0.001	1.000
Mechanism of the injury				
Traffic accident (in the car)	12 (38.7%)	12 (38.7%)	< 0.001	1.000
Traffic accident (pedestrian)	11 (35.5%)	11 (35.5%)	< 0.001	
Fall	6 19.4%)	6 19.4%)	< 0.001	
Invert	0 (0.0%)	0 (0.0%)	< 0.001	
Stab wound/cutting	2 (6.5%)	2 (6.5%)	< 0.001	
Others	0 (0.0%)	0 (0.0%)	< 0.001	
Injury to the admission to the ED				
0–30 (min)	6 (19.3%)	6 (19.3%)	< 0.001	0.963
30–60 (min)	18 (58.0%)	18 (58.0%)	< 0.001	
60–90 (min)	4 (12.9%)	3 (9.67%)	− 0.102	
90– (min)	3 (9.67%)	4 (12.9%)	0.102	
Revised trauma score	5.03 (4.09–6.90)	5.68 (4.45–6.38)	0.086	0.750
Probability of survival	0.684 (0.276–0.878)	0.724 (0.275–0.886)	0.043	0.894
Vital signs on the admission to ED				
Heart rate (per min)	94 (68–120)	105 (80–120)	0.119	0.578
Glasgow coma scale	6 (3–13)	7 (4–14)	0.271	0.228
Systolic Blood Pressure (mmHg)	112 (91–150)	100 (70–140)	− 0.135	0.383
Respiratory rate (per min)	19 (16–24)	24 (16–30)	0.459	0.105
Injury severity score	30 (21–36)	34 (25–41)	0.353	0.182
AIS head and neck	4 (0–5)	4 (0–5)	0.016	0.895
AIS face	0 (0–0)	0 (0–1)	− 0.403	0.414
AIS chest	3 (0–4)	3 (0–4)	− 0.164	0.699
AIS abdomen	0 (0–2)	0 (0–3)	0.660	0.087
AIS extremity	1 (0–3)	2 (0–3)	− 0.295	0.668
AIS external	1 (0–1)	1 (0–1)	0.419	0.678
Blood gas analysis				
pH	7.33 (7.24−7.37)	7.25 (7.15−7.35)	− 0.283	0.095
Base deficit (mmol/L)	3.7 (2.1− 6.7 )	6.3 (2.4–12.5)	− 0.343	0.130
Lactate (mmol/L)	4.1 (2.9–5.5)	4.9 (3.0–7.9)	0.219	0.260
Laboratory data				
Platelet (× 10^3^/μL)	208 (163–240)	193 (114–235)	− 0.158	0.559
PT-INR	1.08 (1.03–1.27)	1.20 (1.10–1.40)	− 0.158	0.078
Fibrinogen (mg/dL)	169 (124–200)	164 (138–219)	0.113	0.647
FDP (μg/mL)	120.0 (66.6–267.0)	93.5 (43.0–188.0)	0.082	0.391
D-dimer (μg/mL)	67.5 (46.5–207.7)	56.9 (20.4–125.0)	− 0.019	0.240

*FC* fibrinogen concentrate, *ED* emergency department, *AIS* Abbreviated Injury Scale, *PT-INR* prothrombin time-international normalised ratio, *FDP* fibrin/fibrinogen degradation products

**Fig. 2:**
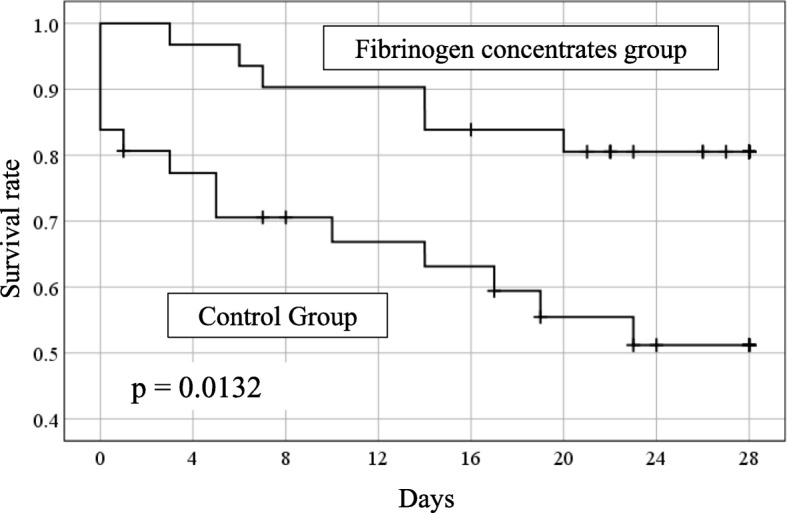
Kaplan-Meier curves for the fibrinogen concentrate (FC) and control groups

Figure 2 shows the matched groups’ Kaplan-Meier survival curves. The FC group had a significantly higher in-hospital survival rate (log-rank *p* = 0.013) and a significantly lower 28-day in-hospital mortality rate (6/31 patients [19.3%] vs. 14/31 patients [45%], *p* = 0.03). During the first 28 days, 16% of patients in the FC group (5/31 patients) had died owing to a brain injury, which was not significantly lower than the 32% rate in the control group (10/31 patients). The rates of haemorrhage-related deaths in the FC and control groups were 0% (0/31 patients) and 6% (2/31 patients), respectively.

Table 3 shows the haemostatic treatments and transfusion requirements in the matched groups, which revealed that the two groups had similar frequencies of haemostatic interventions. The two groups had similar amounts of total FC during the first 24 h after ED admission (*p* = 0.96). The FC group had higher transfusion amounts during the first 6 h after ED admission; however, no significant inter-group differences were observed between 6 and 24 h after ED admission.

**Table 3:** Hemostatic treatments and transfusion amounts in the propensity-matched groups

	Control group *n* = 31	FC group *n* = 31	*p* value
Interventions to emergency haemostasis	5 (16.1%)	10 (32.2%)	0.138
Thoracotomy and/or laparotomy	3 (9.7%)	8 (25.8%)	0.096
Transarterial embolization	3 (9.7%)	2 (6.5%)	0.641
Other emergency interventions	10 (32.2%)	14 (45.1%)	0.297
Craniotomy	8 (25.8%) *	9 (29.0%)	0.753
Orthopaedic surgery	2 (6.5%)	4 (9.7%)	0.390
Other	0 (0.0%)	2 (9.7%)	0.151
Transfusion			
During the first 6 h after the admission to ED			
RBC (unit)	2 (0–10)	8 (2–22)	0.016
FFP (unit)	4 (0–10)	14 (4–23)	0.009
PC (unit)	0 (0–0)	0 (0–20)	0.059
From 6 to 24 h after the admission to ED			
RBC (unit)	0 (0–4)	2 (0–5)	0.387
FFP (unit)	0 (0–7)	4 (0–9)	0.133
PC (unit)	0 (0–15)	0 (0–20)	0.771
FC administration during 24 h after the admission to ED	19 (61.2%)	31 (100%)	< 0.001
First FC administration after the admission to ED			
0–1 h	0	31 (100%)	< 0.001
1–3 h	10 (32.2%)	0 (0.0%)	
3–24 h	9 (29.0%)	0 (0.0%)	
Total amounts during 24 h after the admission to ED	3 (3–3)	3 (3–3)	0.96

*A patients who has performed burr hole surgery was included

*FC* fibrinogen concentrate, *ED* emergency department, *RBC* red blood cell, *FFP* fresh frozen plasma, *PC* platelet concentrate

In patients with blunt trauma (*n* = 142), the propensity score matching process ultimately selected 29 patients from each group (Additional file 1: Table S1), and the FC group had a significantly higher survival rate than the control group (*p* = 0.034) (Additional file 2: Figure S1). We additionally analysed patients with severe brain injury (head AIS ≥ 3, *n* = 97). The propensity score matching process ultimately selected 20 patients from each group (Additional file 1: Table S2); the FC group tended to have a higher survival rate than the control group; however, the difference lacked statistical significance (*p* = 0.174) (Additional file 2: Figure S2.).

## Discussion

The present study is the first to indicate that early FC administration (< 1 h after ED admission) may be useful for patients with severe trauma, based on a propensity score-matched analysis. Many studies have indicated that low fibrinogen levels at ED arrival are associated with haemostatic impairment, massive bleeding, and poor outcomes in patients with severe trauma [11–14, 16, 24–28]. Therefore, early fibrinogen supplementation will help manage trauma-induced coagulopathy [16–22].

Fibrinogen levels deteriorate faster than other haemostatic components during the early phase of severe trauma [6, 11, 27, 29], and early fibrinogen supplementation is crucial for maintaining haemostatic function [16]. In this context, two RCTs have examined the feasibility of early FC administration [39, 42]. Nascimento et al. performed a single-centre RCT that examined FC administration within 50 min after ED admission of patients with severe trauma, and concluded that this approach helped increase plasma fibrinogen levels; however, they acknowledged the need for larger RCTs [42]. However, a second multi-centre RCT examined FC administration within 45 min after ED admission in patients with severe trauma, and found that this approach was not feasible as only 69% of the patients received the intervention within 45 min (versus an intended proportion of 90% of patients receiving the early intervention) [39]. Interestingly, both trials were granted a waiver for obtaining informed consent by the relevant ethics committees [39, 42]. However, if an RCT is planned to evaluate the effects of early FC administration for patients with severe trauma, the same non-consent process may not be approved in other regions including Japan. The present study revealed that early FC administration can easily be performed within 1 h, and was associated with a favourable survival rate after severe trauma in a real clinical setting. Moreover, the FC and control groups had used similar total amounts of FC during the first 24 h after ED admission; however, delayed FC administration (i.e. at 1–24 h after ED admission) was not associated with the same improvement in severe trauma outcomes.

Several previous reports have indicated that FC administration provides various advantages in patients with severe trauma [18, 38, 40, 41]; however, those studies did not specifically examine the time point(s) for FC administration. In our centre, FFP is mainly used for the supplementation of coagulation factors in patients with severe trauma. In addition, FC can be used before starting the FFP administration and/or to boost the fibrinogen levels during FFP administration. Therefore, we evaluated the effects of early FC administration in this setting, which revealed fairly clear advantages for this early treatment strategy.

Although the difference was not significant, we observed that the FC group had approximately one-half the number of brain injury-related deaths in the control group. Furthermore, on sub-group analysis of patients with severe brain injury, although there were no significant differences, we observed that early FC administration tended to improve the survival rate of the patients. In patients with severe brain injury, hyperfibrinolysis is frequently observed at admission to the ED [43, 44]; this contributes to enlargement of intracranial haematomas, trauma-induced coagulopathy, and poor outcomes [43, 45–47]. In this context, FC may help restore haemostasis by complementing plasma fibrinogen, which deteriorates owing to hyperfibrinolysis in patients with severe brain injury. Therefore, FC supplementation may suppress intracranial haematoma enlargement and reduce the risk of death owing to severe brain injury.

In situations with severe bleeding, Geeraedts et al. have suggested that “blind” coagulation management (without point-of-care guidance, such as thromboelastometry) underestimates the real demand for coagulation factors [48]. However, based on the severe decrease in plasma fibrinogen levels during the early phase of severe trauma, we empirically administer FC based on trauma severity alone, before confirming the laboratory test results. Thus, we intentionally “overestimate” the demand for fibrinogen; this is in contrast with the finding reported by Geeraedts et al., who stated that this approach naturally underestimates demand. Although Schöchl et al. have reported the utility of point-of-care guidance [32], the use of FC and its timing in the present study were totally dependent on the attending physicians’ discretion. Therefore, although our “blind” coagulation management using FC without point-of-care guidance may “overestimate” the demand for FC in severe trauma cases, we were still able to effectively administer FC earlier than if we had relied on point-of-care guidance.

In the present study, the FC group had significantly higher amounts of transfusions during the first 6 h after ED arrival, although the FC and control groups had similar total amounts of transfusions (RBC, FFP, and PC) between 6 h and 24 h after ED arrival. Furthermore, the patients in FC group were more recently treated than those in the control groups (this data has not been presented). Therefore, the higher transfusion amounts during the first 6 h and recent advanced treatments may have affected the survival rate in the FC group. Nevertheless, we speculate that the FC may have helped prevent early trauma-related deaths, which may have increased the total need for transfusions in the FC group, thereby introducing the so-called “survival bias”.

The present study has several limitations. The most important limitation is the small number of patients enrolled. The second is the single-centre retrospective study design; however, all eligible patients had available data regarding all variables from ED admission and before FC administration. Third, although we used propensity scores to balance the groups’ characteristics, some variables remained imbalanced. However, it is important to note that most imbalanced variables were more severe in the FC group (vs. the control group); this suggests that FC may have improved the survival ratio even in relatively severe cases. Fourth, the demand for transfusions during the first 6 h of admission to the ED was increased in the FC group. We considered that this result may have been affected by two possible causes, namely, survival bias and recent advances in trauma care. This limitation has been mentioned previously. Therefore, a multi-centre RCT is needed to address these limitations; a planned RCT aiming to identify the optimal timing and dose of fibrinogen supplementation during trauma resuscitation will be of particular value [33].

## Conclusions

The present study revealed a favourable survival rate after early FC administration in patients with severe trauma. In this setting, FC may be an ideal early treatment for managing trauma-induced coagulopathy and may help improve patient outcomes.

## Supplementary information

**Additional file 1: Table S1.** Characteristics of the propensity score matched patients with blunt trauma. **Table S2.** Characteristics of the propensity score matched patients with severe traumatic

**Additional file 2: Figure S1.** Kaplan-Meier curves for the fibrinogen concentrate (FC) and control groups in the pair-matched blunt trauma patients. **Figure S2.** Kaplan-Meier curves for the fibrinogen concentrate (FC) and control groups in the pair-matched severe head trauma patients

## Data Availability

All relevant data are presented in the published manuscript.

## Abbreviations

AIS: Abbreviated Injury Scale
FC: Fibrinogen concentrate
ED: Emergency department
RBC: Red blood cell
FFP: Fresh frozen plasma
PC: Platelet concentrate
