# Supplementary material for: Early administration of fibrinogen concentrate is associated with improved survival among severe trauma patients: a single-centre propensity score-matched analysis
Source: World J Emerg Surg. 2020 Jan 14;15:7. doi: 10.1186/s13017-020-0291-9 (PMC6961302; doi:10.1186/s13017-020-0291-9)
Supplement: Supplementary file 2 — Additional file 2: Figure S1. Kaplan-Meier curves for the fibrinogen concentrate (FC) and control groups in the pair-matched blunt trauma patients. Figure S2. Kaplan-Meier curves for the fibrinogen concentrate (FC) and control groups in the pair-matched severe head trauma patients [file 13017_2020_291_MOESM2_ESM.pptx]

## Slide 1
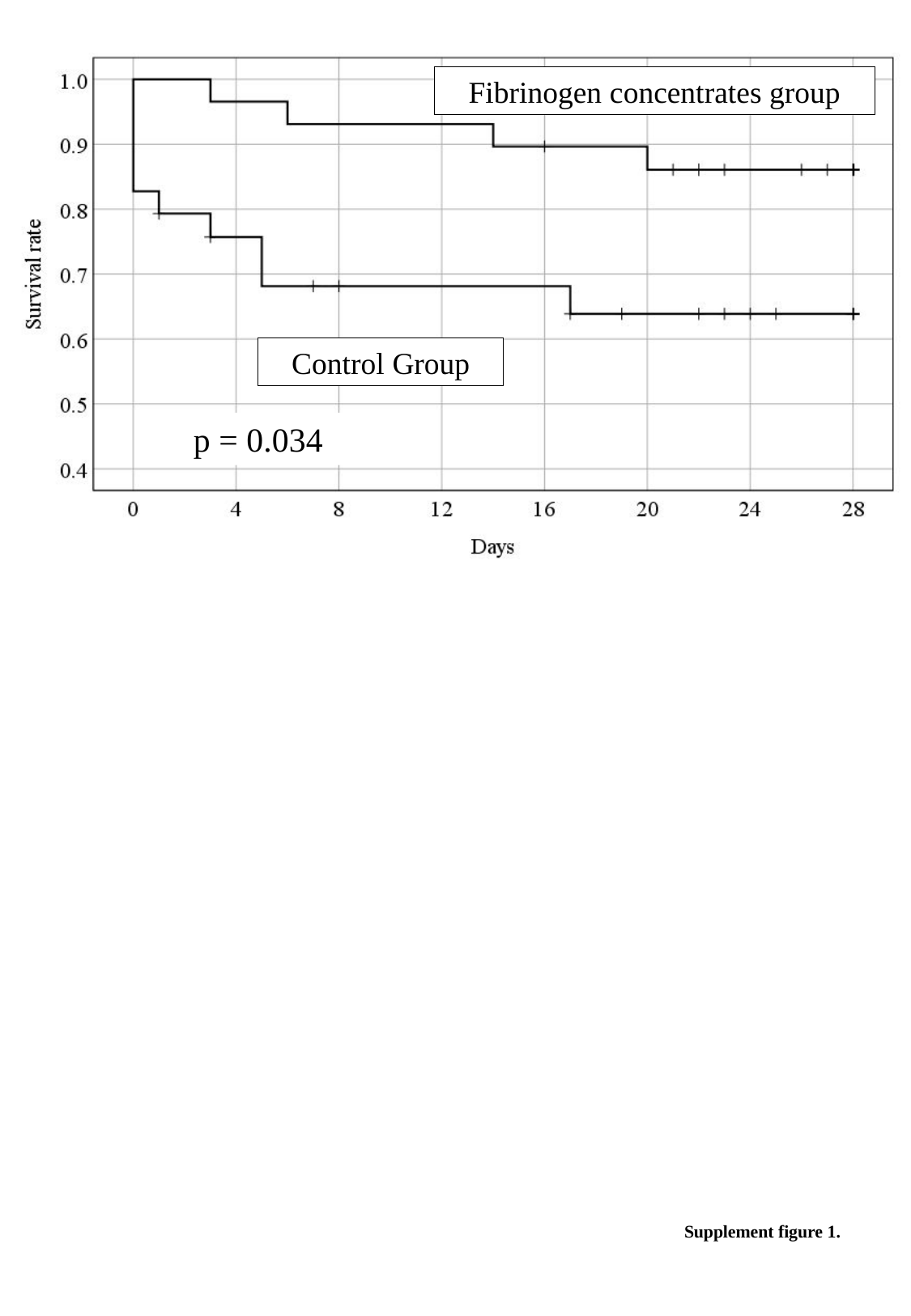

Fibrinogen concentrates group
#
Control Group
p = 0.034
Supplement figure 1.

## Slide 2
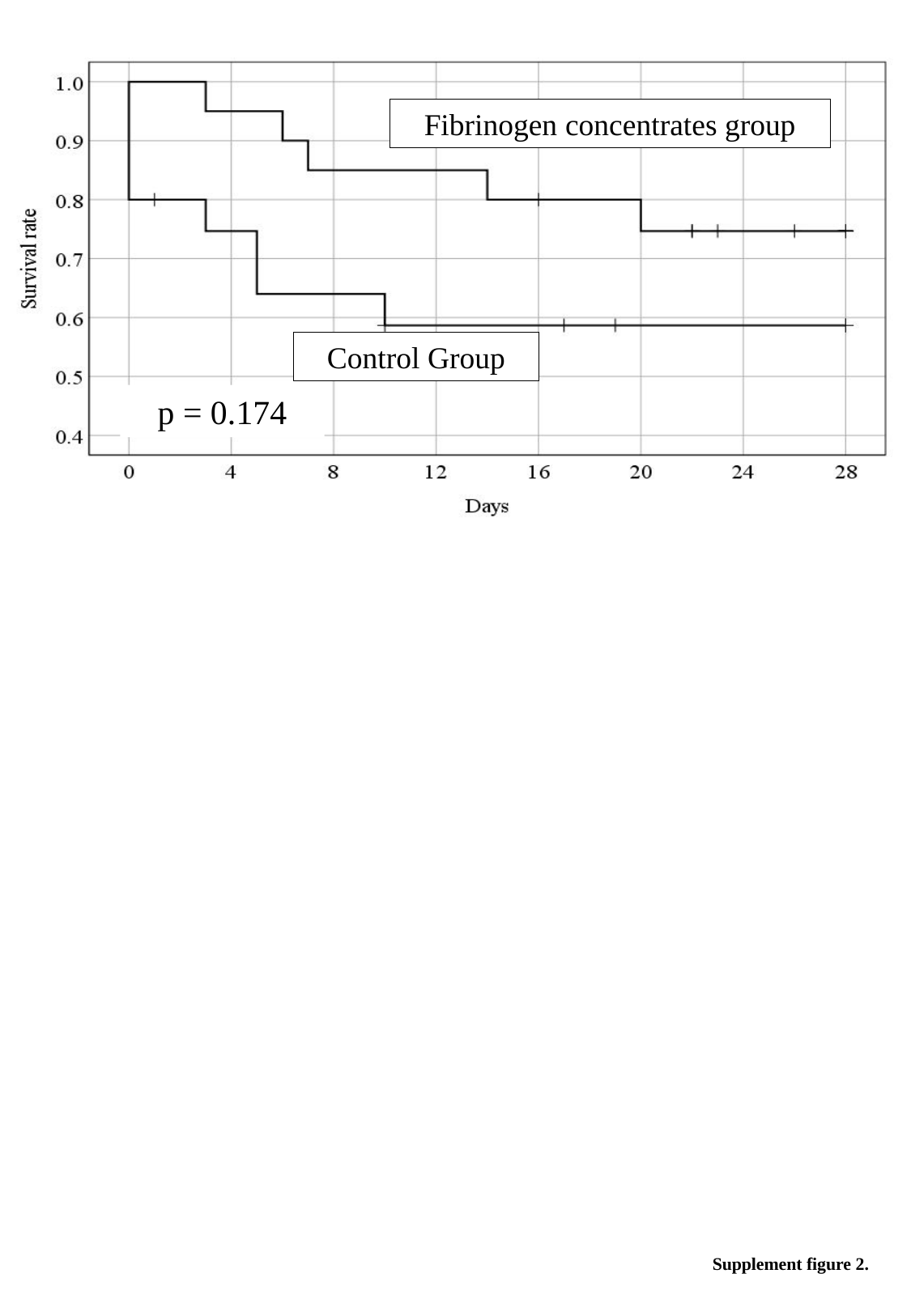

#
Fibrinogen concentrates group
Control Group
p = 0.174
Supplement figure 2.
